# Key actions towards the sustainable management of European geese

**DOI:** 10.1007/s13280-017-0903-0

**Published:** 2017-02-18

**Authors:** David A. Stroud, Jesper Madsen, Anthony D. Fox

**Affiliations:** 1 Joint Nature Conservation Committee, Monkstone House, City Road, Peterborough, PE1 1JY UK; 2 Department of Bioscience, Aarhus University, Kalø, Grenåvej, 8410 Denmark

**Keywords:** Air-strike risk, Conflict resolution, Conservation policies, Crop damage, Ecosystem impacts, Human–wildlife conflict

## Abstract

Increasing abundance of geese in North America and Europe constitutes a major conservation success, but has caused increasing conflicts with economic, health and safety interests, as well as ecosystem impacts. Potential conflict resolution through a single, ‘one size fits all’ policy is hindered by differences in species’ ecology, behaviour, abundance and population status, and in contrasting political and socio-economic environments across the flyways. Effective goose management requires coordinated application of a suite of tools from the local level to strategic flyway management actions. The European Goose Management Platform, established under the Agreement on the Conservation of African-Eurasian Migratory Waterbirds, aims to harmonise and prioritise management, monitoring and conservation efforts, sharing best practice internationally by facilitating agreed policies, coordinating flyway efforts, and sharing and exchanging experiences and information. This depends crucially upon adequate government financing, the collection of necessary monitoring data (e.g., on distribution, abundance, hunting bags, demography, ecosystem and agricultural damage), the collation and effective use of such data and information, as well as the evaluation of outcomes of existing management measures.

## Introduction

The improvement in the conservation status of many European goose populations since the 1940s is one of the major success stories of European bird conservation. Indeed, in many respects, actions to improve the status of these, and other, waterbird species have led the development of effective avian conservation more generally. Fox and Madsen (2017) document the historical development of policies and mechanisms which have contributed to population recoveries from their former depleted status. These included the creation of international conservation organisations such as the International Wildfowl Research Bureau (IWRB, now Wetlands International) (Kuijken 2006), and the development of international legislative frameworks such as the Ramsar Convention in 1971 (Matthews 1993), the European Union’s Directive on the conservation of wild birds in 1979 (‘Birds Directive’; Temple-Lang 1982) and more recently the Agreement on the Conservation of African-Eurasian Migratory Waterbirds (AEWA; Boere 1991, 2010).

The response of migratory goose populations to their coordinated conservation has been dramatic. Since the 1940s, the conservation status of most (although not all) European goose populations has markedly improved, as outlined by Fox and Madsen (2017), in many instances returning them to favourable conservation status. At first, numbers consolidated within newly established nature reserves and other forms of refuge areas (van Roomen and Madsen 1992), but subsequently they have expanded rapidly into agricultural landscapes (Fox and Abraham 2017). As problems associated with these expanding populations developed, the limitations of existing general conservation frameworks to deal with emerging conflict have become apparent, leading initially to increasingly wider scale regional or national policy responses (for the Netherlands: Anon 1990; for Scotland: Scottish Executive 2000; Bainbridge 2017). Yet overall these responses have been largely piecemeal and generally ineffective at reducing conflicts for more than short periods at any location. In part in response to such failures, a ground-breaking population-based international framework (using adaptive management approaches adopted effectively in North America) has been developed as a new way to address the conflicts created by the Svalbard-nesting population of the pink-footed goose *Anser brachyrhynchus* (Madsen et al. 2017).

Meanwhile, many other populations continue to increase and show significant range expansion (e.g. Balmer et al. 2013 for Britain and Ireland), some of which have developed resident populations among formerly migratory species, while others have shifted into new habitats (such as urban environments). Several populations show little sign of strong density dependence, as evidenced by reduced doubling times in numbers, and many now have access to almost unlimited sources of food in contemporary agricultural landscapes (Fox and Abraham 2017). All these trends suggest that, without intervention, the current levels of conflict will, with a high degree of confidence, continue and in all likelihood spread in extent.

However, before turning to addressing some possible solutions to these issues, it is helpful to briefly summarise the impacts of expanding goose populations on other interests.

## Current conflicts

### Conflicts with agriculture

The history of crop protection from geese has developed (Table 1) from essentially simple ‘self-help’ responses by individual farmers aimed at protecting specific vulnerable fields through scaring, to increasingly complex measures to manage goose distribution at ever larger spatial scales, including the creation of refuge (or the so-called Go/No Go) areas within which geese are either tolerated or encouraged, sometimes through the use of sacrificial crops and/or using coordinated scaring (e.g. Koffijberg et al. 2017; Baveco et al. 2017). Whilst such refuge areas have often been successful in the short term, two major factors operate against their long-term success. The first has been the inconsistency in the underlying policy frameworks, as witnessed, for example, in Scotland (McKenzie and Shaw 2017; Bainbridge 2017) and the Netherlands (Koffijberg et al. 2017). The second has been the ever growing increase in goose population sizes. Ultimately, approaches to limit agricultural impacts have had to address the formal need to control abundance at population scales, as has been implemented in North America for greater snow geese *Chen caerulescens atlanticus* (Lefebvre et al. 2017), lesser snow geese *C. c. caerulescens* (Batt et al. 2006) and in Europe for the Svalbard population of pink-footed geese (Madsen et al. 2017).

**Table 1:** Responses to goose damage to farmland at varying scales

Scale of intervention	Type of intervention	Methods used	Implementing agent	Problems	Some case-studies
Local	Scaring from sensitive locations (fields/crops)	Gas guns, flags, streamers, scarecrows, kites, active scaring etc.	Individual farmer	Typically rapid habituation by geese at any location	van Roomen and Madsen (1992) and Fox et al. (2017)
Local	Provision of sacrificial crops	Crop planting, change of cropping	Individual farmer	Cost to establish; attraction of geese, spill-over to adjacent farmland	Fox et al. (2017)
Regional (sub-national)	Displacement from sensitive to less sensitive areas	Creation of disturbance free refuge areas, typically (but not always) accompanied by disturbance in other areas	Groups of farmers, conservation agencies or other stakeholders	Locally can be successful but ultimately gives no constraint on population growth	van Roomen and Madsen (1992), Bignal et al. (1991), McKenzie and Shaw (2017), Eythórsson et al. (2017), Koffijberg et al. (2017) and Simonsen et al. (2017)
Regional to national	Wider scale financial compensation for economic losses or subsidies to allow geese	Financial payments (usually linked to other interventions)	State agencies	Financially unsustainable for growing populations	Anon (1990), van Paassen (1992) and Bainbridge (2017)
Regional to national	Regional population limitation	Legislative change; Adaptive Harvest Management	States and their agencies	Agreement on objectives and target levels; creation of adaptive harvest policy cycle including monitoring	McKenzie (2014), McKenzie and Shaw (2017), Bainbridge 2017 and Lefebvre et al. (2017)
International	Biogeographic population limitation	Legislative change; Adaptive Harvest Management	Multiple states and multilateral environment agencies	Agreement on objectives and target levels; creation of adaptive harvest policy cycle	Batt et al. (2006), Madsen and Williams (2012), Lefebvre et al. (2017) and Madsen et al. (2017)

### Conflicts with other biodiversity

Whilst the impacts of growing goose populations on other biodiversity has been studied on snow geese breeding areas, outside North America and on staging and wintering areas, there has been less research. Buij et al. (2017) review the current knowledge of such ecosystem impacts, showing that impacts on other species can arise not just directly through changes to habitat composition but also indirectly via changes to the physical structure of habitats. They note that negative impacts on natural environments increase particularly when formerly migratory geese become year-round residents (as for barnacle geese *Branta leucopsis* in many European countries), and/or where birds occur in significantly higher densities than traditionally occurred as a consequence of use of farming landscapes.

### Conflicts with air traffic

**Fig. 1:**
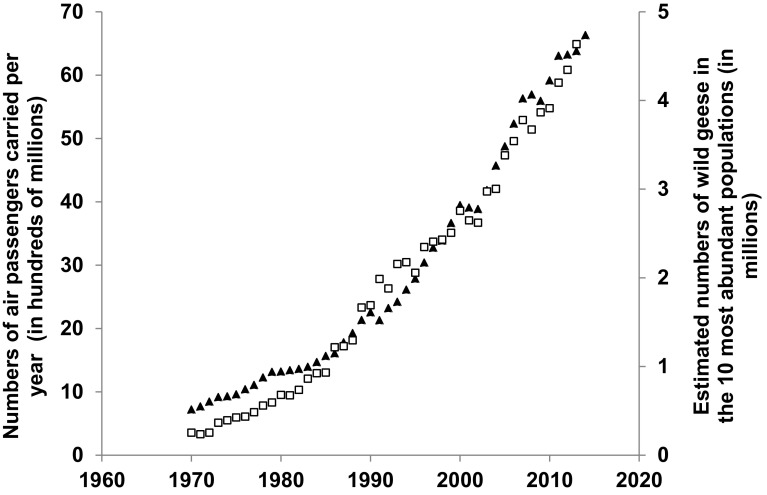
Annual total of air passengers carried in flights from 26 European states, 1970–2014 (*triangles*), compared with the estimated European annual combined abundance of ten most numerous wild goose populations (*squares*—three populations of barnacle geese *Branta leucopsis*, dark-bellied brent geese *B. bernicla bernicla*, Nordic greylag goose *Anser anser*, tundra bean goose *A. fabalis rossicus*, two populations of pink-footed geese *A. brachyrhynchus* and two populations of greater white-fronted geese *A. albifrons*) for 1970–2013. Source for air passengers: World Bank, see http://data.worldbank.org/indicator/IS.AIR.PSGR/countries/EU?page=1&display=default

The growing potential risk of air strikes between planes and geese arise from long-term increases not only of goose numbers, but also from the very significant increase in the air-traffic industry in recent decades (Bradbeer et al. 2017; Fig. 1). Risk is high during the approach, landing and take-off phases of air flights and when geese co-occur in the airspace of airports, although this risk can be reduced by a range of interventions (Bradbeer et al. 2017). However, industry forecasts project a 50% growth in European air transport from 2012 to 2035 (Eurocontrol 2016) which—coupled with projected increases goose populations—suggests that the overall level of risk will not lessen.

## Why current policies/approaches will likely be ineffective in the future

Continued implementation of past responses, whether undertaken at local or regional scales alone, has a high likelihood of being ineffective in the long term. Fundamentally, this is because most geese have shown the behavioural and ecological flexibility to adapt to feeding on modern agricultural landscapes from their former use of natural and comparatively nutrient-poor habitats (Owen 1976). Given that farmland landscapes suitable for goose feeding are effectively unlimited in Europe, future increases in both abundance and range of goose populations can be expected in the immediate future as long as agriculture continues as at present (Fox and Abraham 2017). At present, there are few signs of strong density dependence among many currently increasing populations, and North American experience suggests that it is unlikely that limitation on breeding areas will constrain population growth of geese breeding on European tundra. Furthermore, species which were previously believed to be highly adapted arctic breeders, in particular barnacle geese, have shown an amazing plasticity and capacity to expand their breeding ranges to sub-Arctic and temperate regions (van der Jeugd and Kwak 2017), again escaping potential mechanisms for density dependence.

Because of agreement about their poor conservation status in the 1940s, there has been a common agenda for the conservation of European geese (for example, through the implementation of policies for refuge creation and regulation of hunting), which was essential to restore the favourable conservation status of many goose populations. In contrast, there have been no attempts to date to develop similar coordinated international policies in relation to issues related to wide-scale conflict reduction. Indeed, in the 1990s, the effect of differing national policies in some parts of northern Europe was actually to exacerbate local conflicts as geese redistributed themselves in response to quite different management regimes on different sides of national borders, as was the case for pink-footed geese (Madsen and Jepsen 1992).

Owen (1992), however, saw this need and explored the need for international cooperation with respect to crop damage by geese, calling for a European management plan, which he considered should include the following:

- “provision for detailed monitoring of numbers, breeding success and mortality so that trends and declines below some threshold or ‘safe limit’ can be swiftly detected
- strategic provision of safe roosts and feeding areas throughout the population’s traditional range, and management of local populations so that they use alternative feeding areas rather than farmland
- sensitive control of hunting and shooting under licence in relation to population trends and absolute numbers”.

Twenty-five years on, these same key elements remain just as relevant; yet the need is more pressing than ever.

## Lessons from other situations

In an attempt to provide a framework for best practice, we here summarise some of the key elements that characterise successful interventions, drawn from the studies reported in this special issue and elsewhere.

### Inclusion and transparency

The presence and abundance of geese are significant to many elements of society. For the hunting community, they represent a renewable, recreational resource (which locally can have economic significance). To farmers, geese can be the source of adverse economic impacts. Geese have considerable cultural and aesthetic importance for the public and are often important for the birdwatching community in particular. Geese may locally represent an important source of income from tourists/visitors. Elsewhere, geese may represent a source of risk for those responsible for managing air safety. For conservationists charged with the conservation and wise-use of wetland species and habitats, geese represent important ‘flagship’ taxa. Finally, governments are bound under international legal obligations for the effective conservation and management of goose populations.

Clearly, all these stakeholders have a legitimate stake and important roles to play in decisions to be made about the future development of goose populations. Achieving consensus on population management goals will be challenging given the array of disparate perspectives held by the diversity of interests of such stakeholders. Nevertheless, all must be a part of the process, even if ultimately the end point is a compromise state of ‘least mutual unhappiness’ (Bainbridge 2017).

Batt et al. (2006) highlighted the key importance of early engagement with opinion formers as a critical element of developing a cross-sectoral consensus on management goals for overabundant North American snow geese. Such a tactic has long been recognised as an important element of other types of natural resource conflict resolution (e.g. Kemf 1993). Indeed, the tools and mechanisms to build cross-sectoral engagement and consensus with regard to national resource management are long established (Hesselink et al. 2007) and well known. Such procedures need to be adopted in this context too: there is a critical need to avoid ‘reinventing wheels’. Indeed, the case of overabundant geese is not inherently unusual as a wildlife conflict, other than perhaps that their annual long-distance migrations make them a shared resource which introduces international dimensions to the issue, as well as the dramatic rapidity with which (within one human generation) most geese have recovered from endangered status to now cause a wide range of difficult problems for society.

### Clear initial goal setting to guide processes

Successful management is aided by explicit statements of goals and specific measurable objectives in terms of the ultimate desired state to be achieved. This may differ from target population size which may be subject to changes as a result of adaptive processes.

### Solutions need to be science-based

The most successful solutions are those that are underpinned by sound science and are processes that are systematic and transparent. Modelling in particular can be important in allowing the exploration of potential management scenarios on a more objective basis.

### Adaptive management means long-term organisational commitments

Interventions with an adaptive character allow objectives and actions to be modified and refined on the basis of experience, sometimes repeatedly. However, adaptive solutions are inherently long term and require sustained commitments both politically and financially. Unlike some conservation issues where solutions to problems can be rapid once decisions are made or policy changed, adaptive management of widespread and numerous populations requires continuing organisational/financial commitment (including long-term political support as necessary and appropriate). This also means a commitment to monitoring at appropriate scales (below). The types of data needed for adaptive management processes are outlined by Madsen et al. (2015a, b), who stress that, ultimately, quite simple information can be used in support. Lack of data is not, in itself, a fundamental impediment to progress.

### Solutions ultimately need to be at scale of biological populations

There has been a long history of attempted resolution of goose conflict issues in Europe (as summarised by van Roomen and Madsen 1992). Previous initiatives have been usually local (e.g. the creation of refuge areas to draw birds from sensitive areas), or sometimes through regional or national policies (Table 1). Yet, inherently all such solutions will ultimately fail if populations continue to increase. Thus, as was recognised in North America for snow geese, any solution ultimately needs to be at the scale of the entire biological population.

### Other wildlife conflicts can give lessons

Geese are not the only animals to cause conflicts with human interests, and there is a long history of initiatives to resolve such problems, extensively documented (e.g. Thompson et al. 2010). Inasmuch people and their attitudes (which can either facilitate or impede solutions) are central to most problems, there is much to be learnt from the management of other wildlife conflicts. For example, a review of EU-funded initiatives to resolve problems of coexistence with large carnivores (Silva et al. 2013) provides multiple lessons that are relevant in the context of goose overabundance, for example, organising effective stakeholder engagement and dialogue, communicating strategically and effective working in cross-border situations.

### Solutions need to be coordinated across multiple scales and jurisdictions

The most successful responses typically operate at multiple governance scales, involving several types of intervention (Table 1). Such coordination needs to continue and to be enhanced. The coordinated national and regional delivery of adaptive management plans for relevant populations will facilitate this. It is beneficial to develop strategies not just with ‘top-down’ inputs from senior decision makers (within government and elsewhere), but also including ‘bottom-up’ inputs from those directly affected or who are delivering management on the ground. Interactions between these scales can lead to robust outcomes. The AEWA International Species Management Plan for the Svalbard pink-footed goose is a good example of this (Madsen et al. 2017).

## International legal requirements

Solutions to problems need to conform to international legal frameworks. For Europe, there are three legislative instruments of primary relevance to goose conservation.

The EU Directive on the conservation of wild birds (2009/147/EC) provides the overarching framework for bird conservation within the EU. It requires that “Member States shall take the requisite measures to maintain the population of the species referred to in Article 1^1^ at a level which corresponds in particular to ecological, scientific and cultural requirements, while taking account of economic and recreational requirements, or to adapt the population of these species to that level.^2^” Article 7 of the Directive allows the hunting of species but not at levels that would “jeopardise conservation efforts in their distribution area.” Article 9, however, provides a mechanism for control of species for a range of purposes and following a series of tests and justifications as outlined by European Commission (2008).

The text of the Convention on the conservation of European wildlife and natural habitats (or Bern Convention) is closely aligned to that of the Birds Directive reflecting their common derivation (Lyster 1985). Together, they extend a conceptually single regulatory framework for birds not just across to EU Member States but to Council of Europe Parties also.

AEWA is a stand-alone treaty within the general ambit of the Convention on Migratory Species. In contrast to the Birds Directive, many of its legal provisions are expressed at the scale of populations rather than species (Table 2). The issue over ‘overabundant’ geese was recognised by the fifth Meeting of the Parties in November 2015 which, in Resolution 6.4 (AEWA 2015a), recognised “the need for a coordinated management approach to the Barnacle Goose (*Branta leucopsis*) as well as other goose species in Europe, particularly those with overabundant populations” and requested “the establishment of a European multispecies Goose Management Platform and process to address sustainable use of goose populations and to provide for the resolution of human-goose conflicts, targeting as a matter of priority, Barnacle (*Branta leucopsis*) and Greylag (*Anser anser*) Geese populations for which management plans are yet to be developed as well as the Svalbard population of the Pink-footed Goose (*Anser brachyrhynchus*) and the Taiga Bean Goose (*Anser fabalis fabalis*) for which plans are already in place.” It also invited “interested Parties, Range States and other stakeholders to engage pro-actively in this initiative…”

**Table 2:** Legal status of European goose populations under both the EU Birds Directive (2009/147/EC) and AEWA. Birds Directive: taxa listed on Annex I require the classification of Special Protection Areas under Article 4; Annex IIA indicates the taxon may be potentially hunted in all Member States and IIB only in certain listed Member States (although for all Annex II taxa Member States may chose to nationally restrict hunting). AEWA’s Action Plan status indicates legal quarry status (AEWA 2015b)

Species and race	Population	Birds Directive Annex I	Birds Directive Annex II	AEWA Action Plan
*Branta bernicla bernicla*			Annex IIB	B2b
*Branta bernicla hrota*	Svalbard/Denmark and UK		Annex IIB	A1c
*Branta bernicla hrota*	Canada and Greenland/Ireland		Annex IIB	A3a
*Branta leucopsis*	East Greenland/Scotland and Ireland	Annex I		B1
*Branta leucopsis*	Svalbard/South-west Scotland	Annex I		A3a
*Branta leucopsis*	Russia/Germany and Netherlands	Annex I		C1
*Branta ruficollis*		Annex I		A1a, A1b, A3a, A3c
*Anser anser anser*	Iceland/UK and Ireland		Annex IIA	C1
*Anser anser anser*	NW Europe/South-west Europe		Annex IIA	C1
*Anser anser anser*	Central Europe/North Africa		Annex IIA	B1
*Anser anser rubrirostris*	Black Sea and Turkey		Annex IIA	B1
*Anser fabalis fabalis*	North-east Europe/North-west Europe		Annex IIA	A3c*
*Anser fabalis rossicus*	West and Central Siberia/NE and SW Europe		Annex IIA	C(1)
*Anser brachyrhynchus*	East Greenland and Iceland/UK		Annex IIB	B2a
*Anser brachyrhynchus*	Svalbard/North-west Europe		Annex IIB	B1
*Anser albifrons albifrons*	NW Siberia and NE Europe/North-west Europe		Annex IIB	C1
*Anser albifrons albifrons*	Western Siberia/Central Europe		Annex IIB	C1
*Anser albifrons albifrons*	Western Siberia/Black Sea and Turkey		Annex IIB	C1
*Anser albifrons flavirostris*		Annex I	Annex IIB	A2*
*Anser erythropus*	NE Europe and W Siberia/Black Sea and Caspian	Annex I		A1a, A1b, A2
*Anser erythropus*	Fennoscandia	Annex I		A1a, A1b, A1c

* indicates that a population, otherwise protected, may be hunted on a sustainable use basis within the framework of an international species action plan. This shall seek to implement the principles of adaptive harvest management

A follow-up inter-governmental meeting has since mandated the establishment of the European Goose Management Platform (EGMP; AEWA 2016).

Formal population control at biogeographical population level for species is relatively novel in the context of the Birds Directive—although AEWA’s international adaptive management plan for Svalbard pink-footed goose (Madsen et al. 2017) is being implemented by three Member States, and adaptive harvest management is already embedded with AEWA’s Action Plan (AEWA 2015b), which gives legal obligations for the European Union as a Contracting Party. Article 2 of the Directive, however, clearly indicates an adaptive goal with respect to overall conservation status: that is, the adaption of a species population to a level that “corresponds in particular to ecological, scientific and cultural requirements, while taking account of economic and recreational requirement…”.^3^ Also of significance is that Article 2 relates to “all species of naturally occurring birds in the wild state….”, i.e. it is inclusive of those listed in Annex I. Thus, the Birds Directive provides no legal impediment to the control of species through an adaptive harvest management framework (and/or other policies) in fulfilment of national obligations under Article 2.

## The way forward

The problems outlined above are complex, and both operate and interact at multiple geographic and political scales. Differences in species ecology, behaviour, abundance and population status, as well as in contrasting political and socio-economic environments prevailing across the flyways mean that these problems cannot be tackled through a single, ‘one size fits all’, policy.

Within a single country, the management of goose-related conflicts will be influenced by the implications of the (different) responsibilities of separate central government ministries; relationships between central and devolved (sub-national or provincial) governments; and the interactions between varied government agencies related to the differing issues. All of these essentially concern the question of who has political and financial responsibility for the problem and its solution. Overlain is the issue of communicating and engaging with the public, and of ensuring that state and non-governmental actors share common perspectives, especially since many non-government conservation organisations may be important land-owners and opinion formers, who will also need to manage the perspectives of their members.

There are further issues related to the implications of different national policies between countries, although legislative frameworks such as AEWA and the EU Birds Directive already provide the means for international joint decision making.

Given this complexity, to have any success, it will be essential to develop coordinated and integrated approaches that are mutually supportive. Without such coordination, there is a real risk that groups of interested stakeholders will resort to draconian measures, at risk to the interests of other stakeholders and the long-term stability of populations. Further, actions without a sound scientific basis or by one group of stakeholders may actually exacerbate problems for others. There is a clear need to tackle the problem at the scale of whole populations, integrating the needs and perspectives of all stakeholders in a single process but with the interventions occurring at a range of scales, from local to more strategic, flyway management actions.

The European Goose Management Platform (AEWA 2016) provides a means to deliver these needs but will be critically dependent on adequate funding and political support from the governments of AEWA Parties and other international actors such as the European Commission.

## Practical constraints to rational decision making

There are a number of real world impediments for implementing science-based adaptive population management for European geese. Understanding these can help minimise their significance. These include the following:

- Not all Range States for the populations concerned have the same level of political or administrative engagement with AEWA. Some Range States have yet to ratify AEWA, whilst although some others are AEWA Contracting Parties, they have very low levels of international engagement as expressed by (lack of) submission of national reports and/or attendance at triennial Conferences of the Parties. This will variably affect the national political appetite to engage with the European Goose Management Platform (EGMP) process—especially if there are costs involved (below).
- The governance processes of some countries are significantly compartmentalised. Thus, typically, air-strike risks are dealt with by the transportation ministry, grazing impacts by the agricultural ministry, and species conservation by the environment ministry. Whilst good national governance would suggest that different ministries would develop a common national policy view on cross-cutting issues, this is not always the case.
- A further practical problem relates to funding new mechanisms where the source of funding derives from one ministry but the financial advantages accrue to another sector. Thus, funding for the EGMP will be sought from the budgets of AEWA administrative authorities within national environment ministries but resulting actions will reduce the costs or otherwise benefit other stakeholders. At a time when austerity is being exercised by many European governments, it can be anticipated that there will be reluctance to spend conservation budgets to solve what are seen as agricultural and other problems.
- At a time of reductions, or at least financial constraint, in public funding for bird monitoring programmes, the development of new data-gathering mechanisms in support of the work of an EGMP will be very challenging in many countries.
- Many of the Range States concerned have federal systems of governance such that responsibility for the implementation of environmental (and other) legislation is devolved to sub-national levels. This gives a further level of necessary coordination within states to achieve coherent national policies and processes.

In themselves, none of these problems are insurmountable, but they are likely to result in practical impediments to the initial development of an effective EGMP, not least the speed at which this can be established and develop.

## Recommendations for future actions

Participants^4^ at the international conference on goose management held in Denmark from 27 to 29 October 2015 were asked to provide a prioritised list of actions that would help in response to, and management of, abundant geese. Table 3 synthesises those recommendations together with issues highlighted from the reviews in this special issue.

**Table 3:** Recommendations from the international conference on goose management, Denmark 2015. **An action that is planned (in whole or part) for relevant species through the operation of the European Goose Management Platform (EGMP). (Note that the list is not in priority order)

Recommended actions	For delivery by	Action also relevant to
**Knowledge: actions to improve scientific and other knowledge**		
Develop a common framework for assessing favourable conservation status and setting favourable reference values/target population levels at different scales	European Commission (EC); national authorities	Control; International
Develop advice on simple population modelling for use in data-poor situations	EGMP	Control
Collate better information on migratory routes and population structures of relevant species to support population modelling including coordinated population-wide counts at appropriate frequencies	Wetlands International Goose Specialist Group (GSG); national monitoring schemes	Control
Collate and analyse better data on productivity and other demographic factors, including from marked birds, to aid population modelling	GSG; national and regional monitoring schemes and study groups; EGMP	Control
Agree and promote common methodological standards to facilitate data sharing and joint analyses, and enhance availability of relevant open source data and information	GSG; research organisations; EGMP	
Promote greater research co-operation to avoid duplicative studies	GSG; research organisations; EGMP	
Involve the farming community in scientific studies and research including targeting them in the regular dissemination of derived information	Research organisations; farming stakeholders; EGMP	Stakeholders
Analyse the relationship between population size and crop damage to develop better methods for assessing, and metrics for reporting, ‘serious’ damage for use in management schemes	Research organisations; EGMP	Mitigation
Collect and share data on actual yield losses using standard methodologies	Agricultural authorities; research organisations; EGMP	Mitigation
Promote long-term monitoring of the condition of natural habitats used by geese at all times of the year	Research organisations; EGMP	
**Mitigation and management: actions related primarily to better mitigation and management of existing impacts**		
Review which elements (including socio-economic factors) result in successful measures to prevent/reduce crop damage, especially over multiple years at the same locations	Research organisations	Knowledge
Regularly collate and exchange experience, information and case-studies from different countries including especially examples of failed or ineffective measures, and any cross-border cooperative initiatives	Research organisations; national authorities; EGMP	
Critically review and reconsider those mitigation methods which provide alternative food sources (including sacrificial crops) which then contribute to further population growth	Management authorities; research organisations	
Undertake research on how to make natural habitats more attractive	Management authorities; research organisations	Knowledge
Further develop effective scaring tools including those which result in the aversive conditioning of geese	Research organisations	Knowledge
‘Re-package’ and make more accessible the considerable existing guidance which exists on damage limitation techniques	Management authorities; research organisations	
**Control: actions related primarily to population control using adaptive management measures**		
Promote better engagement with the hunting community, especially the critical need to report, collate and disseminate bag data at all scales (local, national, international), targeting especially those countries where bag data do not exist, or is not readily accessible	National authorities responsible for hunting regulation; hunting organisations; EGMP	Knowledge, Stakeholders
Implement and learn from further examples of practical adaptive management and use this experience to optimise adaptive harvest models	EGMP; national authorities	Knowledge
Review national legislation in relevant countries to ensure its suitability for potential adaptive management processes	National authorities	
Harmonise legal frameworks for the control and management of non-native goose species	National governments	
**Stakeholders: actions related primarily to working with stakeholders**		
Better manage and interact with senior decision makers and politicians to ensure they are asking the right questions, understand the options (including risks and consequences of adaptive management), and have the right information to arrive at decisions	Governmental administrations at all scales; stakeholders	Stakeholders
Make more widely available basic conflict resolution tools and skills with training for conservation professionals and others involved in conflict situations	National conservation agencies and others	
Frequently disseminate relevant information to the public and other stakeholders at multiple scales (international to local)	National conservation agencies; EGMP	
Remove perverse incentives acting against sustainable solutions and replace with incentives appropriately targeted at farmers, hunters and conservation organisations that are mutually supportive	National and regional governments as appropriate	
Produce accessible guidance about the full range of management options related to resolving goose conflicts, and disseminate to policy makers and other stakeholders	National authorities; EGMP; EC	
**International: actions related primarily to international processes**		
Develop and implement flyway-level management plans for relevant populations, based on adaptive management principles, that include: • nested flyway and national management objectives; • a framework for setting complimentary local objectives; • flyway-wide hunting bag limits/targets; • clear statements of monitoring needs; and • thresholds for emergency interventions resulting from dramatic population increases	National governments; EGMP**; EC and Member States; research organisations and other stakeholders	
Establish a better high-level European political vision for goose conservation and management that supports flyway management plans	National governments; EGMP**; EC	Mitigation; Stakeholders; Control
Promote better networking by communicating ‘who does what’ in each country through web-based platforms	EGMP**	
Clarify relationships and the decision-making autonomy between management authorities where, within a country (and especially for those with federal governance), multiple agencies have responsibility for different aspects of goose conservation and management	National authorities at all scales of government	
Produce an overview of the different national policies for compensation and hunting legislation to facilitate development of adaptive management processes	National governments and EGMP	Control
Consider options to revise the EU Birds Directive’s Annex II list of quarry species to aid adaptive management of relevant geese	EC with EU Member States	
Elaborate further existing guidance regarding the interpretation of Article 9 of the Birds Directive (European Commission 2008), which permits derogation from certain of its provisions, in the context of management options for geese	EC	Control
Ensure management of ‘overabundant’ geese does not jeopardise the current favourable conservation status of species concerned, and clarify and agree biologically ‘safe’ population sizes (that accord with favourable conservation status) at national and flyway scales as well as within EU and relevant national legal contexts	National governments; EC and Member States; scientific stakeholders including EGMP	Control

In moving towards adaptive management and the potential stabilisation (or reduction) of some populations, there will be important communication needs. Delivering many of the recommendations in Table 3, whether engaging at high political levels within governments or seeking ‘buy-in’ from the farming community and other stakeholders, necessitates important communication skills. It will be important that the EGMP gives due emphasis to awareness raising activities.

The various conflict situations—despite decades of management interventions—are not diminishing. Indeed with projected increases in numbers, conflicts are likely to continue to grow, potentially very rapidly. A step-change in responses is needed. As indicated by the multiple actors highlighted in Table 3 (from farmers’ organisations to academic researchers, and from conservation organisations to national authorities), solutions need to be delivered by many, working together to what must be shared objectives.

Finally, Owen (1992), in urging international cooperation to address crop damage in Europe, concluded “I consider planning to be preferable to inaction.” We agree.

## Footnotes

1 i.e. all species of naturally occurring birds in a wild state.

2 Article 2.

3 However, as Lyster (1985) noted, in such adaptation, any economic and recreational requirements are clearly subservient to ecological, scientific and cultural requirements, otherwise the Directive would have simply stated *“ecological, scientific, cultural, economic and recreational requirements…”.*

4 Participants included national and regional government representatives, managers and experts from Belgium, Canada, Denmark, Estonia, France, Iceland, the Netherlands, Norway and the UK as well as relevant international organisations.
